# Supplementary material for: How does digital transformation affect the ESG performance of Chinese manufacturing state-owned enterprises?—Based on the mediating mechanism of dynamic capabilities and the moderating mechanism of the institutional environment
Source: PLoS One. 2024 May 14;19(5):e0301864. doi: 10.1371/journal.pone.0301864 (PMC11093376; doi:10.1371/journal.pone.0301864)
Supplement: Attachment — Submitted filename: plosone.docx [file pone.0301864.s002.docx]

Manuscript title: How does digital transformation impact corporate ESG performance？ Evidence from China's state-owned manufacturing enterprises

Manuscript ID:PONE-D-23-43453

**Summary of the paper:** This paper studies the relationship between digital transformation and ESG performance. Using a sample of Chinese listed firms, the authors document that digital transformation is an important factor in promoting the improvement of ESG performance of state-owned enterprises. The authors further investigate the mechanism of digital transformation from the perspectives of dynamic capabilities and institutional environment. These findings can provide empirical evidence for improving ESG performance and promoting corporate sustainability. The paper concentrates on an important issue about ESG performance in the context of emerging economics or developing countries. In addition, the moderating effects of institutional environment influence ESG practices of enterprises in the long-run. The authors highlight different interactive governance mechanisms for digital transformation and how they influence the ESG practices of Chinese listed firms. This paper examines an interesting question and obtains supporting evidence. However, there are a few comments and concerns on the current draft. I suggest to the authors only minor revisions:

1. Relationship to the literature and contribution:The discussion of contributions lacks reference support. A more nuanced discussion of extant research and their outcomes would be necessary to contextualize the contribution of the paper. For example, in line 94-106: The authors state that ”explores the mechanism of digital transformation from the perspectives of institutional environment and dynamic capability....."Are these mechanism analysis concepts widely used by related literature? The description lacks literature support,which might confuse the first contribution with the second.The paper would benefit greatly if the authors could point out more clearly in the introduction section that how exactly this paper provides novel insights into the literature.

2. Research hypothesis :Line 145-148:The authors state that “The performance of corporate social responsibility mainly emphasizes that enterprises should strengthen the governance of the relationship between multiple stakeholders....."This may seem applicable to all enterprises(including SOE and non-SOE). The authors should emphasize that this situation is unique to state-owned enterprises and update the whole paragraph.

3.Line 165-186:The authors hypothesize that: “The corporate governance performance of an enterprise includes two dimensions: the internal governance and the external governance performance....”in Section 1.1 . However, moderating role of Formal institutions and Informal institutions are highlighted again in Section 1.3. It will mislead readers to confuse on the real mechanism of digital transformation. In addition, H2 and H3 are proven by moderating effects,which is different from dynamic capabilities mechanism.

4.Theoretical framework:It is recommended to reshape the theoretical framework and add a diagram or figure to help readers quickly understand the whole story and core research variables.

5. Robustness Tests:The robustness test should display the related table on page21.It is very clear that the authors emphasize Digital Transformation have positive effect on Corporate E,S and G in section2. The paper would benefit greatly if the authors could show the different impacts of digital transformation on three aspects of ESG.

6.Other comments:

The formula in line 428 and 433 is not labeled.

The hypothesis order is incorrect.

There are some grammar mistakes. The author should conduct grammar checks and proofreading

There is a wealth of literature on digital transformation and ESG. The authors should cite English literature rather than Chinese.
